# Performance of cervical phIGFBP-1 test alone or combined with short cervical length to predict spontaneous preterm birth in symptomatic women

**DOI:** 10.1038/s41598-017-11447-y

**Published:** 2017-09-07

**Authors:** Florent Fuchs, Marie Houllier, Soizic Leparco, Anne Guyot, Marie-Victoire Senat, Hervé Fernandez

**Affiliations:** 1 Department of Obstetrics and Gynecology. Hôpital Bicêtre, Assistance Publique Hôpitaux de Paris (APHP), Le Kremlin-Bicêtre, France; 2 Inserm, CESP Centre for research in Epidemiology and Population Health, U1018, Reproduction and child development, Villejuif, France; 3 Faculté de Médecine Paris-Sud, Université Paris-Sud, 94276 Le Kremlin-Bicêtre, France

## Abstract

We aimed to assess the accuracy of cervical phosphorylated insulin-like growth factor binding protein-1 (phIGFBP-1) test alone or in combination with cervical length (CL), to predict preterm birth (PTB) in symptomatic women. We performed a prospective cohort study from 2012 to 2015 including singleton pregnancies with symptoms of preterm labor, intact membranes and CL < 25 mm at 24–34 weeks of gestation. Studied outcome were spontaneous delivery within 7 and 14 days of testing and spontaneous PTB at <34 and <37 weeks of gestation. Among 180 women, 21 (11.7%) had a positive phIGFBP-1 test. Spontaneous PTB occurred within 7 days, 14 days of testing and before 34 weeks and 37 weeks in 7.8%, 10.6%, 12.9% and 28.8%, respectively. The phIGFBP-1 test had a low predictive performance for all studied outcomes varying for positive likelihood ratios (2.8 to 3.4) and negative likelihood ratios (0.8). Combining phIGFBP-1 and CL did not increase its predictive ability. After adjustment, positive phIGFBP-1 test was no more independently associated with a delivery within 7 days (p = 0.55), unlike CL < 15 mm (p = 0.04). In conclusion, phIGFBP-1 test alone or in combination with CL has a low predictive accuracy to predict PTB in symptomatic women.

## Introduction

Preterm birth, defined as the birth of a child before 37 weeks of gestation, is one of the most important determinants of neonatal morbidity and mortality. About 15 million preterm births occur each year in the world with a variable incidence of 5–18%^1^. In France, preterm births, which represent 7.4% of all births, are responsible for 30% of cerebral palsy, thus constituting a major public health problem^2^. Indeed, despite the advances in neonatal resuscitation, the rate of neurological complications, respiratory complications and infectious complications has not declined for several years. Besides, preterm birth is also associated with an increased risk of long-term neurodevelopmental impairment among surviving babies, including cerebral palsy, that are directly related to the gestational age at birth^3, 4^. About two-thirds of preterm births (PTB) are spontaneous, following spontaneous onset of labor or premature rupture of the membranes, whereas the remaining third is medically indicated because of maternal or fetal complications^5^.

In attempt to reduce the risk of spontaneous PTB, health professionals have tried to find reliable diagnostic tests to better target asymptomatic patients at risk, but also and mainly to predict PTB among symptomatic patient. Reliable prediction of PTB could allow interventions to delay birth in patients with true preterm labor and also avoid the use of unnecessary and costly interventions such as hospitalization in patients with false preterm labor.

One of these tests is the Actim Partus test (cervical phosphorylated insulin like growth factor binding protein-1 (phIGFBP-1)). PhIGFBP-1 is a protein synthesized in decidualized endometrium cells during pregnancy and that is absent in the vagina under normal condition. Under uterine contractions, leakage of phIGFBP-1 into the vagina car happen^6^. Therefore, its presence in vaginal secretion might indicate an increased risk of PTB among patients with symptoms of preterm labor. Several authors have reported the accuracy of cervical phIGFBP-1 among symptomatic patients, but mainly among small cohort (<100) with heterogeneous inclusion criteria (singletons, twins, short cervical length…) or in comparison with other predictive tests (fetal fibronectine, cytokines…). Results of these studies were recently summarize in a systematic review and metaanalysis that conclude to an overall low to moderate predictive accuracy of phIGFBP-1 and pointed out the need for large well-design prospective studies in symptomatic women^7^.

The objective of our study was to evaluate the predictive accuracy of phIGFBP-1 for spontaneous PTB in a large prospective study of singleton pregnancies with symptoms of preterm labor and a short cervical length, and to compare it with the use of cervical length measurement alone or contingently.

## Materials and Methods

This is a prospective cohort study performed in a French tertiary care center from September 2012 to August 2015. All women older than 18 years with a singleton pregnancy between 24 and 34 weeks gestation with symptoms of preterm labor, intact membranes and a short cervical length (<25 mm) were included in the study after providing informed consent. Preterm labor was defined by the presence of regular uterine contractions, lasting at least 30 seconds and occurring at least three times per 10 minutes, associated with significant cervical changes during transvaginal sonographic examination (cervical length <25 mm). Non-inclusion criteria were confirmed rupture of membranes, cervical dilatation >3 cm, cervical length ≥25 mm at ultrasound, prolapse membranes bulging in the vagina, cervical cerclage, vaginal bleeding, placenta previa, placental abruption, severe intrauterine growth restriction, fetal malformation and preeclampsia. Exclusion criterion was medically indicated preterm delivery.

Women admitted with symptoms of preterm labor had first a transvaginal sonographic measurement of cervical length according to standard protocol (empty bladder, minimal pressure, no ultrasound gel applied over the probe protection, measurement of the shortest length between the internal and external os, with clearest image after 3 measurements, before and after valsalva manœuvre)^8^. If the cervical length was <25 mm, the patient could be included in the study. All included women followed the same protocol. Each patient was first examined with a vaginal speculum to check for closing of the cervix or opening of the cervix with membranes at external os or presence of a prolapse amniotic sac bulging in the vagina. A first swab was inserted in the vagina to exclude premature preterm rupture of membranes (Actim Prom, MEDIX BIOCHEMICA). Then, a second swab was rotated in the posterior fornix of the vagina and sent to the laboratory for bacteriological analysis. A third swab (Actim Partus, produced by MEDIX BIOCHEMICA before year 2013 and by ALERE after year 2013) was taken in the cervix and held for 15 seconds, then dipped into a sterile medium and held for another 10 seconds. Following this, a dipstick was used to determine if the test was positive (two blue lines), suggesting a concentration of phIGFBP-1 in the cervical secretions higher than 10 mg/L, or negative (single blue line after 5 minutes). The midwife in charge of the patient performed all swab tests, but it was another midwife from another department who performed the analysis of phIGFBP-1 test and reported the result in a masked file, blinding it for both the midwife and the obstetrician in charge of the patient. The patient was also blind to the result of the test. Vaginal swab testing were carried out either on admission or within 24 hours of admission if a digital examination had been performed in the 24 hours before the patient’s inclusion in the study. In summary, patients and caregivers were blinded to the result of phIGFBP-1 test (double blinded). The patient was then admitted to the high-risk pregnancy department, underwent blood and urinary cytobacteriological tests, received administration of tocolytics for 48 hours, corticosteroids, and prescription of bed rest according to local protocol. Demographic, maternal and fetal characteristics were recorded. Gestational age was defined according to first trimester ultrasound scan.

Outcomes studied were spontaneous delivery within 7 and 14 days of testing and spontaneous preterm birth at <34 and <37 weeks of gestation. For each outcome studied (<7 days, <14 days, <34 weeks, <37 weeks) we excluded patients with preterm delivery due to induction of labor (except for preterm premature rupture of membranes (pPROM) that were kept in the study) or preterm elective cesarean delivery.

First, a descriptive analysis of the population was carried out. For variables whose distribution was normal, the results are presented in mean +/− standard deviation [extreme] otherwise, the median and the 1st and 3rd quartiles are provided.

Univariate analysis on the performance of phIGFBP-1 test in predicting the different outcomes was performed. Then, we carried out a comparison of the diagnostic performance (sensitivity, specificity, positive and negative predictive value, positive and negative likelihood ratio) of short cervical length <15 mm and positive phIGFBP-1 testing for the same issues. A combine analysis of cervical length and phIGFBP-1 was also performed: This combination test was considered positive either if cervical length was <15 mm or if cervical length was 15–24.9 mm with positive phIGFBP-1. The combination test was considered negative if cervical length was 15–24.9 mm with negative phIGFBP-1. Likelihood ratios for a positive test result above 10 and likelihood ratios for a negative test result below 0.1 are considered to provide strong prediction. Moderate prediction can be achieved with likelihood ratios of 5–10 and 0.1–0.2, whereas those <5 and >0.2 give only minimal prediction^9^.

Finally, as suggested by Conde-Agudelo and Romero in their metaanalysis^7^, we studied the risk factors for delivery within 7 days through univariate and multivariate analysis using multiple logistic regression that included variables with significance level less than 0.20 in the previous univariate analyzes.

Statistical analysis was performed with STATA v.13 software (Stata Corporation, College Station, TX). Our manuscript follows the STARD criteria for diagnostic accuracy studies. All methods used in our study were carried out in accordance with French guidelines. This study received approbation of French ethics committee (CPP Ile de France VII) under the notification number PP 10–006, N° HAO 10012 - NI 10009.

## Results

During the study period of time, 342 patients were referred to the emergency department for symptoms of preterm labor. Among these, 140 had a cervical length ≥25 mm at ultrasound, 20 refused to enter the study protocol and 2 were lost to follow up leaving 180 women entering the study.

Population characteristics, pregnancy outcome and phIGFBP-1 test are presented in Table 1. Women included had a mean age of 28.8 years +/−6.2, where mainly Caucasian (49.2%), had a mean body mass index of 23.5 +/−4.4 at the beginning of pregnancy and smoked cigarettes for 9.4%. Past obstetrical history of these patients revealed history of late (15 to 22 weeks) miscarriage (11.1%), very early and early (22 to 32 weeks) preterm labor in 8.3% or late (32 to 37 weeks) preterm labor in 7.2%. Mean gestational age at inclusion was 30.4 weeks +/−2.6 (range 24.1 to 33.9 weeks) with a mean body mass index of 27.1 +/−4.4 at inclusion. Mean cervical length at transvaginal sonographic examination was 15.3 mm +/−6.4. Distribution of short cervix (<10 mm, <15 mm, and <20 mm) was as followed: 25.6%, 47.2%, and 76.7% respectively. phIGFBP-1 test was positive in 21 patients (11.7%).

**Table 1:** Population characteristics.

Characteristics	N = 180
Maternal age, years (mean +/−SD)	28.8+/−6.2
**Ethnicity**	
Europe and Nord Africa	88 (49.2)
Central and West Africa	70 (39.1)
Asian	12 (6.1)
Indian	4 (2.2)
Other	6 (3.4)
Nulliparous, n (%)	72 (40)
Gestational age at inclusion, weeks (mean +/−SD)	30.4+/−2.6
Cervical length at inclusion, mm (mean +/−SD)	15.3+/−6.4
Cervical length <10 mm, n (%)	46 (25.6)
Cervical length <15 mm, n (%)	85 (47.2)
Positive phIGFBP-1, n (%)	21 (11.7)
Gestational age at delivery, weeks (mean +/−SD)	37.4+/−2.9
Spontaneous delivery <34 weeks, n (%)	23/179 (12.9%)
Spontaneous delivery <37 weeks, n (%)	51/177 (28.8%)
Inclusion to delivery interval, days (median (1^st^–3^rd^ quartile))	48.7 (31.2–66.9)
Spontaneous delivery <7 days, n (%)	14/180 (7.8%)
Spontaneous delivery <14 days, n (%)	19/180 (10.6%)

During hospitalization every patient received administration of tocolytis: Per os Nifedipine tablets alone (56%), Per os Nifedipine tablets followed by Nifedipine LP (9%), Intravenous Nicardipine (27%) or Intravenous Atosiban (8%); and 2 dose of 12 mg of corticosteroids (betamethasone) 24 hours apart. Biological testing at entry revealed high C-Reactive Protein (10%) and bacterial positive vaginal swab in 18.3% requiring antibiotic/antifungal treatment in 22 cases (12.2%).

Mean gestational age at delivery was 37.4 weeks +/−2.9 with a distribution of preterm birth <34 weeks and <37 weeks of 13.3% and 30% respectively. After exclusion of induced prematurity, spontaneous preterm delivery occurred in 23/179 (12.9%) before 34 weeks and in 51/177 (28.8%) before 37 weeks. Median days from testing to delivery were 48.3 days [31.2–66.9] with 14 patients (7.8%) and 19 patients (10.6%) spontaneously delivering respectively within 7 days and 14 days of testing.

Mode of delivery was cesarean (15%) and women gave birth to a male neonates (56%) with a mean birthweight of 2896 g +/−615, an APGAR score < 7 at 5 minutes in 4.4%, an umbilical arterial pH < 7.10 in 4% and umbilical arterial lactates >5 mmol/L in 10%.

Performance of phIGFBP-1 test in predicting the different outcomes is reported in Table 2. A positive phIGFBP-1 test predicted appropriately preterm delivery regardless of studied outcome. The odds of delivering within 7 days of testing was 3.5 (95% CI: 1.1–12.4) with a p value of 0.04. Comparison of the diagnostic performance of cervical length < 15 mm, positive phIGFBP-1 test and positive combination test to predict spontaneous preterm delivery <7 days, <14 days, <34 weeks and <37 weeks is summarized in Table 3. The cervical phIGFBP-1 test had a low predictive performance for all studied outcomes with sensitivities, specificities, positive and negative likelihood ratios that varied between 23.5% and 31.6%, 89.8% and 92.9%, 2.8 and 3.4, and stable to 0.8, respectively. Cervical length <15 mm always had the best negative likelihood ratio (LR) ranging from 0.2 to 0.4 corresponding to a moderate or low accuracy to identify women not at risk to deliver spontaneously within the corresponding outcome. Positive combination test, compared to cervical length <15 mm alone, did not enable to change the predictive accuracy and never reached a better prediction than a low accuracy for predicting preterm delivery (LR +< 5 and LR −> 0.2).

**Table 2:** Risk of spontaneous preterm delivery (<34 weeks and <37 weeks) and risk of spontaneous delivery within 7 days and 14 days of testing, in patients with symptoms of preterm labor and positive cervical phIGFBP-1 testing.

Risk of delivery with positive phIGFBP-1 testing	<7days	<14days	<34 weeks	<37 weeks
p value	0.04	0.004	0.003	0 0.002
OR* (95%CI)	3.5 (1.1; 12.4)	4.5 (1.5; 12.4)	4.4 (1.6; 12.6)	4.1 (1.6; 10.2)

*OR: odds ratio (95% Confidence interval).

**Table 3:** Prediction performance of different diagnostic methods for different outcomes.

	Sensitivity (95% CI)	Specificity (95% CI)	PPV** (95% CI)	NPV^†^ (95% CI)	LR + ° (95% CI)	LR −^‡^ (95% CI)
Delivery <7 days (frequency = 14/180 = 7.8%)						
Cervical length <15 mm	85.7% (57.2–98.2%)	56% (48.1–63.7%)	14.1% (7.5–23.4%)	97.9% (92.6–99.7%)	2.0 (1.5–2.6)	0.2 (0.07–0.9)
ph-IGFBP1+	28.6% (8.4–58.1%)	89.8% (84.1–93.9%)	19% (5.5–41.9%)	93.7% (88.7–96.9%)	2.8 (1.1–7.2)	0.8 (0.6–1.1)
Positive combination test*	92.9% (66.1–99.8%)	51.8% (43.9–59.6%)	14% (7.7–22.7%)	98.9% (93.8–100%)	1.9 (1.6–2.4)	0.2 (0.02–0.9)
Delivery <14 days (frequency = 19/180 = 10.6%)						
Cervical length <15 mm	84.2% (60.4–96.6%)	57.1% (49.1–64.9%)	18.8% (11.2–28.8%)	96.8% (91–99.3%)	2.0 (1.5–2.6)	0.2 (0.1–0.8)
ph-IGFBP1+	31.6% (12.6–56.6%)	90.7% (85.1–94.7%)	28.6% (11.3–52.2%)	91.8% (86.4–95.6%)	3.4 (1.5–7.7)	0.8 (0.5–1.0)
Positive combination test*	89.5% (66.9–98.7%)	52.8% (44.8–60.7%)	18.3% (11–27.6%)	97.7% (91.9–99.7%)	1.9 (1.5–2.4)	0.2 (0.05–0.7)
Delivery <34 weeks (frequency = 23/179 = 12.9%)						
Cervical length <15 mm	82.6% (61.2–95%)	58.3% (50.2–66.2%)	22.6% (14.2–33%)	95.8% (89.6–98.8%)	2.0 (1.5–2.6)	0.3 (0.1–0.7)
ph-IGFBP1+	30.4% (13.2–52.9%)	91% (85.4–95%)	33.3% (14.6–57%)	89.9% (84.1–94.1%)	3.4 (1.5–7.5)	0.8 (0.6–1)
Positive combination test*	87% (66.4–97.2%)	53.8% (45.7–61.8%)	21.7% (13.8–31.6%)	96.6% (90.3–99.3%)	1.9 (1.5–2.4)	0.3 (0.1–0.7)
Delivery <37 weeks (frequency = 51/177 = 28.8%)						
Cervical length <15 mm	72.5% (58.3–81.1%)	63.5% (54.4–71.9%)	44.6% (33.7–55.9%)	85.1% (76.3–91.6%)	2.0 (1.5–2.6)	0.4 (0.3–0.7)
ph-IGFBP1+	23.5% (12.8–37.5%)	92.9% (86.9–96.7%)	57.1% (34–78.2%)	75% (67.4–81.6%)	3.3 (1.5–7.3)	0.8 (0.7–1.0)
Positive combination test*	76.5% (62.5–87.2%)	58.7% (49.6–67.4%)	42.9% (32.5–53.7%)	86% (76.9–92.6%)	1.9 (1.4–2.4)	0.4 (0.2–0.7)

*Positive test if cervical length <15 mm or if cervical length = 15–24.9 mm with positive ph IGFBP1. Negative test if cervical length = 15–24.9 mm with negative ph IGFBP1.

**Positive Predictive value.

°Negative predictive value.

^†^Positive Likelihood ratio.

^‡^Negative Likelihood ratio.

Multivariate logistic regression of risk factors predicting spontaneous preterm delivery within 7 days of testing is presented in Table 4. After adjustment on covariates, phIGFBP-1 positive test was no more independently associated with a delivery within 7 days with an odd ratio of 1.6 (95%CI: 0.3–5.8); p = 0.55. On the contrary, cervical length <15 mm remained independently associated with a delivery with 7 days of testing with odd ratio of 4.3 (95%CI: 1.1–18.6); p = 0.04.

**Table 4:** Multivariate logistic regression of risk factors predicting spontaneous preterm delivery within 7 days of testing.

Risk Factor	OR (95% CI)*	p value
Maternal age		0.76
<25 years	1	
25–35 years	1.7 (0.3–10.7)	
>35 years	2.2 (0.3–18.6)	
History of preterm delivery	1.2 (0.1–14.3)	0.90
C-reactive protein (increased of 1 mg/dl)	1.1 (1.0–1.2)	0.07
Gestational age at inclusion (increased of 1 week)	1.3 (1.0–1.8)	0.08
Open cervix with membranes visible at external os**	16.5 (2.6–105.8)	0.01
Cervical length <15 mm	4.3 (1.1–18.6)	0.04
Positive phIGFBP-1 test	1.6 (0.3–5.8)	0.55

*OR: odds ratio (95% Confidence interval)

**Speculum examination. Membranes at external os but not bulging in the vagina

## Discussion

Our prospective double blind cohort study show that, overall, the cervical phIGFBP-1 test has a low predictive accuracy for preterm birth at <34 and <37 weeks of gestation and for delivery within 7 and 14 days of testing in singleton pregnancies with symptoms of preterm labor and a short cervical length. Even if a positive phIGFBP-1 test is associated with an increased risk of preterm delivery, its predictive performance is lower than other tests such as cervical length <15 mm. When combining phIGFBP-1 test and cervical length measurement together in a two-step sequence; first cervical length measurement and then phIGFBP-1 test if cervical length 15–24 mm; this contingent method does not provide an increased performance prediction, with still low performance. When assessing delivery within 7 days of testing and after adjustment on covariates, a positive phIGFBP-1 test does not independently predict this outcome unlike a short cervical length.

Since the advent of transvaginal cervical length measurement, researches have focused on identifying a novel predictive test or marker than could, among women who present with symptoms of preterm labor, be able to identify those who would deliver within 48 hours to 7 days of presentation in order to rationally guide the administration of antenatal tocolytic agents, corticosteroids and in utero transfer to a tertiary care center. Fetal fibronectine (fFN) was the first bedside test availaible and showed promising results, especially regarding its high negative predictive value^10, 11^. Development of phIGFBP-1 test rose hopeful but its predictive accuracy was discordant according to various studies published^12–14^. The metaanalysis by Conde-Agudelo and Romero^7^ confirmed heterogeneity in the results and pointed out the need for larger well-designed prospective cohort study. Our study retrieved similar results as those mentioned in both metaanalyses with, positive and negative likelihood ratios of phIGFBP-1 test ranging from 2.9 to 4.3 and 0.2 to 0.4 respectively^15^. The only difference was that we had in our study a higher specificity and lower sensitivity that contributed to increase positive LR and decrease negative LR. Our results, based on a large prospective cohort study were disappointed, as phIGFBP-1 was not able to predict with high accuracy a preterm birth even with strict inclusion criterion.

The strengths of our study rely on a rigorous methodology limiting therefore the risks of bias. We included a large cohort of consecutive pregnant women referred to the obstetric emergency department for symptoms of preterm labor. Every woman during the study period was asked to enter the study and we only had a small number of women who declined to participate (5.8%). This low rate of refusal is probably due to the fact that the phIGFBP-1 test is a simple vaginal swab test and that instead of performing 2 vaginal swabs (Actim Prom and bacteriological testing) the research consisted only in a third swab, which is not an invasive procedure. Unlike other studies, the population included followed strict criterion. This study was a prospective cohort study, design to analyze the performance of phIGFBP-1 and not a secondary analysis of another trial^16^. None women had a cerclage performed or carried a uterine malformation that could have increase propensity to deliver earlier^17^. The definition of preterm labor was clearly stated in our study^16, 17^.

As phIGFBP-1 test is qualitative, the risk of patient misclassification was null, unlike studies using quantitative tests carrying the risks to change the cut off value for a positive one. Another strength is that each patient with symptoms of preterm labor was systematically tested against preterm premature rupture of membranes (pPROM) using Actim Prom test. As amniotic fluid is full of IGFBP-1, a false positive Actim Partus test due to pPROM was not possible in our study. Besides no study has ever been published, showing that the use of multiple swabs at the same time could affect the results and the outcome of those tests.

Patients included in the study, as well as the medical team in charge of the patient (midwives, nurses, obstetricians, residents…), was blinded to the result of phIGFBP-1 test thanks to the help from midwives from another department. This blinding was maintained during the study thanks to a masked file. As clinicians were blinded to the test result, they did not apply different protocol to the patients according to their phIGFBP-1 status. A strict use of the local protocol for preterm labor risk was applied. Regarding the outcome, we excluded non-spontaneous deliveries in order to evaluate properly the prediction of the test. Besides, all outcomes had been defined in the study protocol at the beginning of the study.

Our study has some limitations. First, our main limitation is the relatively low incidence of preterm labor in this cohort of symptomatic women with a short cervix (28.8%). Even if we decided to select patients also according to their cervical length, it finally appears that the diagnostic criteria employed for preterm labor in our study were no enough stringent. This would argue that the study population in our paper is much lower risk and therefore may not be comparable to other populations. It may also explain why the test was not found to be predictive of PTB. Therefore our study might be underpowered to show such differences. However, the incidence is consistent with other studies using similar inclusion criteria, and the one we applied corresponded to internationally recommended criteria for PTB^2^. Moreover, we believe that the cervical length’s cut-off we have chosen (<25 mm) is useful to exclude women with false preterm labor, while the cut-off <15 mm is usually useful to predict preterm birth within 7 days. Choosing a more stringent cut-off would have strongly reduce the number of patients and would also have selected a very small sample of high risk patients that do not correspond to those admitted in obstetrical emergency triage room.

We were unable to study the performance of phIGFBP-1 test to predict a delivery with 48 h of testing as only 5 out 180 women (2.8%) effectively delivered within two days and none of them had a positive test. Our analysis was then centered to identify the best predictive method for a delivery within 7 days of testing. Another limitation of our study is that we did not compare phIGFBP-1 test with fFN test. We do not use routinely fFN test and the purpose of our study was to better characterize the performance of this new test (phIGFBP-1) that was presupposed to be better due to its physiology. Finally, the odds ratios presented in Table 4 are quite high, but with wide confidence intervals, which could suggest that our study may have insufficient power to accurately test the study hypothesis. However, univariate analysis and predictive values are in accordance with those previously published in literature.

In conclusion, our study confirmed that phIGFBP-1 test should not be used as a routine bedside test to predict a preterm birth among symptomatic women, as is has only a low predictive accuracy. Other diagnostic tests should now be evaluated in order to predict 2–7 days delivering patients that could benefit the most from hospital admission, tocolysis and corticosteroids. Moreover, the most important is probably also to find a test that predict women who will not deliver between 2–7 days limiting unnecessary transfers and hospital admissions, enabling to reassure the patients.
